# Antimalarial activity of the anticancer and proteasome inhibitor bortezomib and its analog ZL_3_B

**DOI:** 10.1186/1472-6904-7-13

**Published:** 2007-10-23

**Authors:** Jennifer M Reynolds, Kamal El Bissati, Jens Brandenburg, Arthur Günzl, Choukri Ben Mamoun

**Affiliations:** 1 Department of Genetics and Developmental Biology, University of Connecticut Health Center, 263 Farmington Ave, Farmington, CT 06030-3301, USA

## Abstract

**Background:**

The high rate of mortality due to malaria and the worldwide distribution of parasite resistance to the commonly used antimalarial drugs chloroquine and pyrimethamine emphasize the urgent need for the development of new antimalarial drugs. An alternative approach to the long and uncertain process of designing and developing new compounds is to identify among the armamentarium of drugs already approved for clinical treatment of various human diseases those that may have strong antimalarial activity.

**Methods:**

Proteasome inhibitor bortezomib (Velcade™: [(1R)-3-methyl-1-[[(2S)-1-oxo-3-phenyl-2-[(pyrazinylcarbonyl) amino]propyl]amino]butyl] boronic acid), which has been approved for treatment of patients with multiple myeloma, and a second boronate analog Z-Leu-Leu-Leu-B(OH)_2 _(ZL_3_B), were tested against four different strains of *P. falciparum *(3D7, HB3, W2 and Dd2) that are either sensitive or have different levels of resistance to the antimalarial drugs pyrimethamine and chloroquine.

**Results:**

Bortezomib and ZL_3_B are equally effective against drug-sensitive and -resistant parasites and block intraerythrocytic development prior to DNA synthesis, but have no effect on parasite egress or invasion.

**Conclusion:**

The identification of bortezomib and its analog as potent antimalarial drugs will set the stage for the advancement of this class of compounds, either alone or in combination therapy, for treatment of malaria, and emphasize the need for large-scale screens to identify new antimalarials within the library of clinically approved compounds.

## Background

Malaria is caused by intraerythrocytic protozoan parasites of the genus *Plasmodium*. It is responsible for more than 300 million clinical cases and over 2 million deaths annually [1]. *Plasmodium falciparum*, the organism that causes the most lethal form of the disease, is becoming increasingly resistant to almost all available drugs in the antimalarial armamentarium [1]. New chemotherapeutic strategies are therefore urgently needed to combat this disease.

During its intraerythrocytic life cycle, a single *P. falciparum *parasite undergoes multiple morphological and physiological changes and multiplies to produce up to 36 new daughter parasites in ~48 hours. Large-scale genomic and proteomic analyses revealed a coordinated program of gene and protein expression during parasite intraerythrocytic life cycle [2-7]. The first phase of this program occurs during parasite transition from ring to trophzoite stage and is marked by the induction of expression of enzymes required for biosynthesis of proteins and membranes, nutrient acquisition, and degradation of the host cytoplasm. The second phase occurs during transition from trophozoite to early schizont and is manifested by the induction of expression of enzymes required for biosynthesis of ribonucleotides and deoxyribonucleotides and for DNA replication. The third phase occurs during parasite schizogony and is marked by the induction of subunits of the proteasome. The last phase of this program occurs during late schizogony and immediately after invasion and becomes evident by the expression of specific proteins required for host cell invasion [2]. The rise and fall of expression of subsets of proteins during specific stages of parasite intraerythrocytic life cycle suggest a coordinated control of protein turnover during parasite development. In eukaryotes, such regulation is controlled by the proteasome.

Proteasomes are multicatalytic protease complexes whose principle task is the selective degradation of proteins within the cell. Although a fully intact proteasome has not been isolated from *P. falciparum*, the sequencing of this organism revealed a complete set of ORFs encoding homologs of eukaryotic subunits of the proteasome [8-10]. The expression of seven α and six β subunits of the 20S particle and 16 subunits of the 19S regulatory particle of the putative *P. falciparum *proteasome suggest an important role for this multicatalytic complex in parasite intraerythrocytic cycle. Interestingly, this expression peaks during parasite transition from developmental, structural and metabolic functions to more specialized functions important for the generation of new daughter parasites capable of completing the cycle and invading new host cells [5,6]. This suggests that the parasite proteasome could play an important role in protein turnover and parasite replication. Accordingly, the proteasome inhibitor lactacystin was found to inhibit erythrocytic schizogony of *P. falciparum *prior, but not subsequent, to DNA synthesis and parasite multiplication [11].

Several studies have highlighted the importance of proteasome inhibition as a possible approach for the treatment of cancer and parasitic diseases [11-13]. Lindenthal and colleagues showed that the boronate analog MLN-273 blocks the exoerythrocytic development of *P. berghei *and the intraerythrocytic development of *P. falciparum *[12].

Here we provide data indicating that the proteasome inhibitor and analog of MLN-273, bortezomib (Velcade™: [(1R)-3-methyl-1-[[(2S)-1-oxo-3-phenyl-2-[(pyrazinylcarbonyl) amino]propyl]amino]butyl] boronic acid), which has been approved for treatment of patients with multiple myeloma, and a second boronate analog Z-Leu-Leu-Leu-B(OH)_2 _(ZL_3_B), which was found to be highly toxic to trypanosomatid parasites (IC_50 _of 0.32 nM in culture; [14]) are potent inhibitors of *P. falciparum*.

Bortezomib was the first proteasome inhibitor shown to have anti-cancer activity and to induce a marked and durable response in patients with multiple myeloma in clinical trials [15]. We have tested bortezomib and ZL_3_B in different strains of *P. falciparum *including strains that are resistant to pyrimethamine and chloroquine. We found that both compounds are equally effective against drug-sensitive and -resistant parasites with inhibitory concentrations in the low nanomolar range. The compounds block intraerythrocytic development prior to DNA synthesis, but had no effect on parasite egress or invasion.

## Methods

### Strains

The clones 3D7, HB3, Dd2, and W2 of *P. falciparum *used in this study were obtained from the Malaria Research and Reference Reagent Resource Center (MR4).

### Cell Culture and Materials

Parasites were cultured by the method of Trager and Jensen [16] by using a gas mixture of 3% O_2_, 3% CO_2_, and 94% N_2_. RPMI medium 1640 was supplemented with 30 mg/liter hypoxanthine (Sigma), 25 mM Hepes (Sigma), 0.225% NaHCO_3 _(Sigma), 0.5% Albumax I (Life Technologies, Grand Island, NY), and 10 μg/ml gentamycin (Life Technologies). Bortezomib was purchased from the University of Connecticut Health Center Pharmacy. ZL_3_B was purchased from Boston Biochemical Inc. (Cat# I-120). Parasite synchronization was obtained with three successive 5% sorbitol treatments [17]. To determine visually the stage of the parasite life cycle, fixed smears of the *P. falciparum*-infected erythrocytes were stained with Giemsa stain and analyzed by bright-field microscopy.

### Hypoxanthine incorporation assay

The susceptibility of parasites to different compounds was assessed by tritiated hypoxanthine uptake as described by Desjardins and colleagues [18]. Briefly, infected erythrocytes (2% hematocrit, 3% rings) were washed and incubated with the appropriate drugs at the listed concentrations in hypoxanthine-free media for 48 hours. 200 μL of the mixture was then added to a 96 well plate with ^3^H-hypoxanthine at a concentration of 0.5 μCi/well. Following an incubation of 24 hours, the cells were washed on an ultrafilter and radioactivity was counted using a scintillation counter. IC_50_'s are represented in nM. Values are means ± standard deviation of three indipendent experiments each performed in triplicate. These experiments were performed at least three times with similar results.

## Results and Discussion

### ZL_3_B and bortezomib inhibit the *P. falciparum *intraerythrocytic cycle

**Figure 1:**
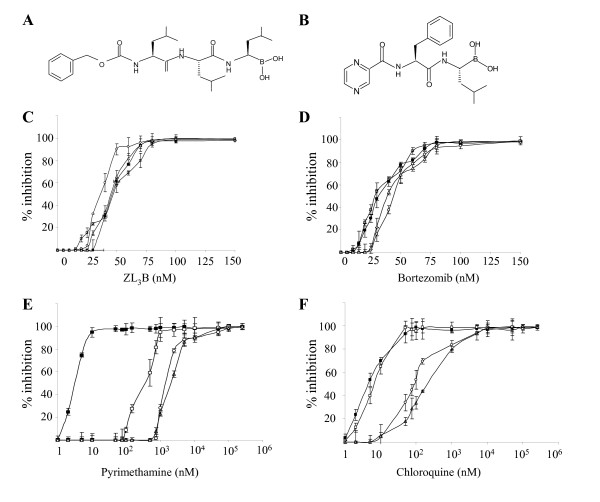
Structures of ZL_3_B (**A**) and bortezomib (**B**). Inhibition of 3D7 (closed squares), HB3 (open squares), W2 (open circles), and Dd2 (open triangles) parasite clones as a function of ZL_3_B (**C**), bortezomib (**D**), pyrimethamine (**E**) and chloroquine (**F**) concentrations. The clones of *P. falciparum *used in this study were obtained from the Malaria Research and Reference Reagent Resource Center (MR4). IC_50_'s are represented in nM. Values are means ± standard deviation of three independent experiments each performed in triplicate.

The cell permeable peptide boronate Z-Leu-Leu-Leu-B(OH)_2 _(ZL_3_B) (Fig. 1A) is a specific and potent proteasome inhibitor that blocks the growth of the bloodstream form of the protozoan parasite *Trypanosoma brucei *with a 50% inhibitory concentration (IC_50_) of 0.32 nM in culture [14]. In order to examine the antimalarial activity of ZL_3_B, we have tested the effect of increasing concentrations of this compound up to 200 nM on the intraerythrocytic life cycle of *P. falciparum *in culture by following the incorporation of radiolabeled hypoxanthine into parasite nucleic acids. The study was performed with four different strains of *P. falciparum *(3D7, HB3, W2 and Dd2) that are either sensitive or have different levels of resistance to the antimalarial drugs pyrimethamine and chloroquine (Fig. 1C–F and Table 1). ZL_3_B was found to inhibit parasite proliferation with an IC_50 _values between 34 and 45 nM. Due to the strong antimalarial activity of ZL_3_B, we speculated that the ZL_3_B analog and clinically approved peptide boronate, bortezomib (Fig. 1B) might have a similar antimalarial activity. Bortezomib is a boronic acid dipeptide and a reversible inhibitor of the chymotrypsin-like activity of the 20S proteasome [15]. It strongly and selectively inhibits the proteasome, has substantial cytotoxicity against a broad range of human tumor cells and has shown excellent anti-tumour activity in preclinical and clinical trials [15]. Bortezomib was approved by the U.S. Food and Drug Administration and the European commission for the treatment of advanced multiple myeloma, and more recently, it received a fast track status for relapsed and refractory mantle cell lymphoma [13]. We first analyzed the antimalarial activity of bortezomib by using the hypoxanthine assay and found that the drug inhibited proliferation of *P. falciparum *3D7, HB3, W2 and Dd2 strains with IC_50 _values ranging between 31 and 43 nM (Fig. 1D and Table 1). As a control, we confirmed the IC_50 _of chloroquine and pyrimethamine in the four strains (Fig. 1E and 1F, and Table 1). As expected, strain 3D7 was sensitive to both compounds with IC_50 _of 6 ± 0.2 and 5 ± 1.1 nM, respectively; strain HB3 was sensitive to chloroquine (IC_50_: 8 ± 1.4 nM) and resistant to pyrimethamine (IC_50_: 500 ± 45 nM); strain W2 was moderately resistant to chloroquine (IC_50_: 90 ± 3.7 nM) and highly resistant to pyrimethamine (IC_50_: 1.5 ± 0.006 μM), and Dd2 was highly resistant to both chloroquine (IC_50_: 300 ± 21 nM) and pyrimethamine (IC_50_: 2.5 ± 0.097 μM). To further confirm the inhibitory effects of bortezomib and ZL_3_B on *P. falciparum *growth, we employed the pLDH colorimetric assay, which measures the production of parasite specific lactate dehydrogenase activity [19-21]. Consistent with the results of the hypoxanthine incorporation assay, both compounds were found to inhibit equally well chloroquine- and pyrimenthamine- sensitive and resistant strains (not shown). Noteworthy, these studies were also consistent with the finding that another boronic derivative, MLN-273, inhibits the intraerythrocytic development of *P. falciparum *[12].

**Table 1:** 50% Inhibitory concentrations IC50 (nM) of ZL_3 _B, bortezomib, chloroquine and pyrimethamine in *P. falciparum *strains

	**3D7**	**HB3**	**W2**	**Dd2**
**ZL_3_B**	40 ± 12	45 ± 5.8	34 ± 3.9	40 ± 11.1
**Bortezomib**	31 ± 1.8	31 ± 2.7	43 ± 4	37 ± 5.1
**Chloroquine**	6 ± 0.2	8 ± 1.4	90 ± 3.7	300 ± 21
**Pyrimethamine**	5 ± 1.1	500 ± 45	1500 ± 5.8	2500 ± 97

### Bortezomib and ZL_3_B antimalarial activities occur prior to DNA synthesis

**Figure 2:**
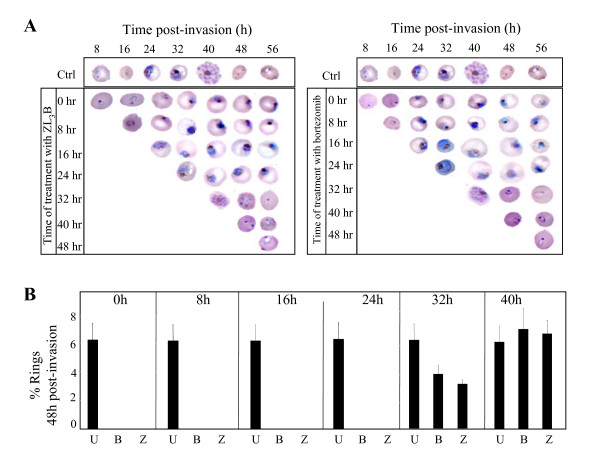
(**A**) Stage specific inhibition of *P. falciparum *(3D7) parasite by ZL_3_B and bortezomib. Highly synchronized cultures of the parasites were grown in the absence or presence of 100 nM of ZL_3_B or bortezomib, stained by Giemsa stain, and analyzed by light microscopy.**(B) **Estimated number of daughter rings formed 48 h following parasite (3D7) invasion of host erythrocytes in the absence (U) or presence of ZL_3_B (Z) or bortezomib (B). Drugs were applied at 8, 16, 24, 32, 40 and 48 h following parasite invasion.

To determine the developmental stage during which bortezomib and ZL_3_B exert their antimalarial effects, *P. falciparum *cultures were synchronized and the proteasome inhibitors were added to the culture medium at different times following parasite invasion and a final concentration of 100 nM. Culture samples were collected every 6 hours and parasite intraerythrocytic developmental progression was monitored by Giemsa staining and light microscopic analysis (Fig. 2A). As a control, an untreated culture of *P. falciparum*-infected erythrocytes was monitored. In the absence of bortezomib or ZL_3_B, the parasite displayed a normal cycle progression from rings to trophozoites, trophozoites to schizonts and schizonts to rings in approximately 44 hours (Fig. 2A). Addition of bortezomib or ZL_3_B during the ring (8, 16 h post-invasion) or early trophozoite (24 h post-invasion) stages resulted in a complete blockage of developmental progression and subsequent death of the parasites. Treatment with these compounds during the late trophozoite stage (32 h post-invasion) only partially blocked parasite progression (Fig. 2B). On the other hand, treatment during the schizont stage (40 h post-invasion) had no effect on parasite progression (Fig. 2A). This stage-specific inhibitory effect of bortezomib and ZL_3_B was quantified by counting the number of rings that developed 48 hours post-invasion in the absence or presence of the compounds. Consistent with the previous analysis, no rings could be detected from cultures treated with bortezomib or ZL_3_B 8, 16 or 24 h post-invasion. In contrast, treatment with these compounds 32 h post-invasion reduced the number of rings in comparison to untreated parasites by 40% only (Fig. 2B) and treatment 40 h post-invasion was completely ineffective (not shown). Together these data suggest that these compounds inhibit parasite development and multiplication and have a lesser effect on the release of merozoites from the infected erythrocytes or on the invasion of new red blood cells by the released merozoites. Although a direct effect of these compounds on the proteasome has not been investigated, our data along with the known mode of action of these compounds in other cell lines suggest an important role for the *P. falciparum *proteasome in parasite development and DNA synthesis.

The recommended adult dose of bortezomib for treatment of myeloma is 1.3 mg/m^2^; and in children the compound is used at a dose of 1.2 mg/m^2 ^[22-24]. The mean peak plasma concentration (Cmax) determined 5 min after drug administration at doses between 1.3 and 1.7 mg/m^2 ^was 63 ± 16 ng/ml and the mean area under the concentration-time curve extrapolated to infinity (AUCinf) was 27 h ng/ml [23]. These values are 2 to 4-fold the IC_50 _observed with bortezomib in *P. falciparum*. *In vivo *studies to determine the dose and tolerability of this compound for treatment of malaria are warranted.

## Conclusion

Our studies demonstrate that two boronates, ZL_3_B and its clinically-approved analog bortezomib, are potent inhibitors of the intraerythrocytic cycle of both drug-sensitive and resistant *P. falciparum *strains. These findings will set the stage for the evaluation of this new class of compounds for treatment and/or prophylaxis of *falciparum *malaria. Furthermore, our studies set the stage for large-scale screens to identify new antimalarials among clinically approved drugs. This approach could shorten the lengthy and expensive process of designing, developing and testing the potency, efficacy and safety of new drugs.

## Competing interests

The author(s) declare that they have no competing interests.

## Authors' contributions

JMR and KEB carried out all the experiments presented in this work and helped in the writing and revision of the manuscript. JB helped in the design of the study and interpretation of the data. AG and CBM conceived and designed the project, helped in the analysis and interpretation of the data, and the writing and revision of the manuscript. All authors read and approved the final manuscript.

## Pre-publication history

The pre-publication history for this paper can be accessed here:


